# Dual inhibition of TGFβ and PDGF improves RV remodeling and function in response to RV pressure or volume‐loading

**DOI:** 10.14814/phy2.70339

**Published:** 2025-05-05

**Authors:** John D. Dauz, Kana Yazaki, Yohei Akazawa, Theo A. Meister, Golam Kabir, Sachiko Kadowaki, Osami Honjo, Scott P. Heximer, Rachel M. Wald, Kim A. Connelly, Mark K. Friedberg

**Affiliations:** ^1^ Institute of Medical Science University of Toronto Toronto Ontario Canada; ^2^ Division of Cardiology, Labatt Family Heart Centre The Hospital for Sick Children Toronto Ontario Canada; ^3^ Li Ka Shing Knowledge Institute Keenan Research Centre, St. Michael's Hospital Toronto Ontario Canada; ^4^ Division of Cardiovascular Surgery The Labatt Family Heart Centre, The Hospital for Sick Children Toronto Ontario Canada; ^5^ Department of Physiology University of Toronto Toronto Ontario Canada; ^6^ Toronto Congenital Cardiac Centre for Adults Peter Munk Cardiac Centre Toronto Ontario Canada

**Keywords:** congenital heart disease, fibrosis, hypertrophy, PDGF, pressure‐loading, right ventricle, TGFβ, volume‐loading

## Abstract

Right ventricular (RV) pressure and volume loading induce RV fibrosis in association with RV dysfunction, morbidity, and mortality in repaired tetralogy of Fallot. Transforming‐growth factor‐β1 (TGFβ1) and platelet‐derived growth factor (PDGF) activate common downstream signaling pathways via TGFβ canonical and non‐canonical signaling to promote increased fibroblast activation, proliferation, and fibrosis in other organs. However, the role of PDGF and TGFβ canonical and non‐canonical signaling in RV fibrosis is incompletely characterized. Here, we investigate whether dual inhibition of TGFβ and PDGF, using Tranilast (TRN), improves RV remodeling in response to pulmonary artery banding (PAB) or pulmonary regurgitation (PR). TRN reduced TGFβ canonical signaling in PAB rats associated with improved RV fibrosis, hypertrophy, and RV function. In response to PR, TRN reduced PDGFRβ expression and normalized ERK1/2 activity, which were associated with reduced RV hypertrophy and improved diastolic relaxation. We identify that PDGF drives RV fibroblast proliferation and activation via SMAD2/3, JNK, and β‐catenin signaling. Our studies suggest that TGFβ and PDGF are interconnected drivers of RV fibrosis and hence synergistic targets to improve RV remodeling in RV pressure and volume loading.

## 1 INTRODUCTION

Advances in the surgical management of congenital heart disease (CHD) have improved patient survival into adulthood (Apitz et al., 2009). Patients with repaired tetralogy of Fallot (rTOF) constitute a large portion of these patients, and many require re‐intervention or are at risk for late morbidity and mortality due to residual lesions such as pulmonary or branch pulmonary artery stenosis (PS) and/or regurgitation (PR) (Apitz et al., 2009). These lesions cause chronic right ventricular (RV) pressure and/or volume loading, respectively, leading to RV dysfunction and ultimately failure from RV hypertrophy, dilatation, and fibrosis (Frigiola et al., 2004; Yamamura et al., 2019). RV dysfunction is an important determinant of late outcomes in CHD and other conditions such as pulmonary arterial hypertension (PAH) which pressure‐load the RV (Driessen et al., 2018). Histological and cardiovascular magnetic resonance imaging markers of RV fibrosis are associated with RV dysfunction and with adverse clinical outcomes in rTOF and other conditions (Cochet et al., 2019; Yamamura et al., 2019). However, the molecular mechanisms underlying RV fibrosis, dysfunction, and ultimately failure remain incompletely understood. Consequently, there is a lack of therapies for RV failure.

Transforming growth factor‐β1 (TGFβ1) and platelet‐derived growth factor (PDGF) are key mediators of fibroblast activation and proliferation, extracellular matrix (ECM) remodeling, and collagen deposition in several organs, including the heart (Frangogiannis, 2021). We have consistently found increased expression of TGFβ1 and PDGF signaling components in rodent models of RV pressure and volume loading, in association with RV hypertrophy, fibrosis, and dysfunction (Akazawa et al., 2021; Ebata et al., 2021; Gold et al., 2020; Nielsen et al., 2016; Ramos et al., 2018; Sun et al., 2018). TGFβ1 binds to its heterodimeric receptor (TGFβRI/II) to promote fibrosis via canonical (SMAD2/3) and non‐canonical (mitogen‐activated protein kinases (MAPKs)) signaling, which includes extracellular signal‐regulated kinase (ERK), c‐Jun NH2‐terminal kinase (JNK), p38 MAPK (p38), and protein kinase B (Akt)) (Frangogiannis, 2021; Khalil et al., 2017). In cardiac fibroblasts, cytoplasmic phosphorylated SMAD2/3 translocate into the nucleus, initiating transcription of profibrotic genes such as COL1A1, COL3A1, Ctgf, Acta2, and Postn (Khalil et al., 2017). TGFβ1 non‐canonical signaling molecules are upregulated in response to stress stimuli in the heart; however, their role in the generation of RV fibrosis is unclear (Rose et al., 2010). PDGF receptors (PDGFRs), and several other receptor tyrosine kinases, have common downstream signaling components with TGFβ1 signaling, so that molecular crosstalk occurs between these pathways (Bonner, 2004). PDGFRs bind to proteins that activate the RAS/MAPK and PI3K/Akt pathways (Bonner, 2004). Akt signaling can further activate Wnt signaling, which is highly upregulated in the pressure‐loaded RV, mediating fibrosis and metabolic changes (Fang et al., 2007; Nayakanti et al., 2023). PDGF‐induced mesenchymal proliferation and collagen deposition are mediated by ERK and p38 MAPKs (Bonner, 2004). However, the relative contribution of and crosstalk between TGFβ1 and PDGF signaling are incompletely characterized in the pressure‐or volume‐loaded RV. Furthermore, the contribution of PDGF to the development of RV fibrosis is not defined.

From a translational standpoint, as TGFβ1 and PDGF signaling can crosstalk and reciprocally enhance one another, their combined inhibition may be synergistically more effective in reducing RV fibrosis compared with targeting either alone (Lee et al., 2016; Zhao et al., 2013). Tranilast (TRN) is a dual inhibitor of TGFβ1 and PDGF which reduces fibrosis in renal and left ventricular (LV) disease (Chen et al., 2021; See et al., 2013; Kelly, 2004; Kelly et al., 2007). However, to our knowledge, the effects of dual TGFβ and PDGF inhibition have not been tested in the hemodynamically stressed RV. To evaluate the effects of combined TGFβ1 and PDGF inhibition on RV remodeling, we administered TRN to rat models of isolated RV pressure‐ or volume‐loading and examined RV histopathology, function, and TGFβ canonical and non‐canonical signaling. To define the role of PDGF in the development of RV fibrosis and the crosstalk between PDGF and TGFβ1 signaling, we examined in vitro the impact of PDGF inhibition (AG1295) on mechanically and TGFβ1‐stressed RV fibroblasts. We hypothesized that in RV pressure‐ and volume‐loading, dual inhibition of TGFβ and PDGF: (1) reduces RV fibrosis and improves RV function; and (2) PDGF inhibition downregulates TGFβ1 canonical and non‐canonical signaling thereby reducing RV fibroblast proliferation and activation.

## 2 MATERIALS AND METHODS

### 2.1 Animal models

Animal experiments were performed in accordance with humane guidelines of the American Physiologic Society and approved by the institutional animal care committee (Protocol #59576). Animals were housed in a temperature‐controlled (22 ± 1°C) environment and 12‐h light/dark cycle. Commercial rat chow (5058, LabDiet, Indiana, USA) and acidified water were provided ad libitum.

#### 2.1.1 Pulmonary artery banding

Male Sprague–Dawley rats (6–7 weeks old) weighing 250 ± 20 g (*n* = 10–12/group) (Charles River, Senneville, Quebec) were randomized to undergo a pulmonary artery banding (PAB) or sham procedure as previously described (Sun et al., 2018). Briefly, anesthesia was induced using 3%–5% isoflurane, and animals were intubated and mechanically ventilated using a volume‐controlled respirator (Kent Scientific, Torrington, CT). Anesthesia was maintained at 2% isoflurane. Following a left thoracotomy, a 16G needle and suture were used to create a fixed constriction in the main pulmonary artery. The needle was removed, and the thorax and skin were closed in layers. Surgical sham controls were generated by performing a left thoracotomy. One week after surgery, animals were started on 300 mg/kg/day TRN (T714570, Toronto Research Chemicals, Toronto, ON) or vehicle (0.5% carboxymethylcellulose), *p.o*., until the terminal experiment 5 weeks post‐surgery (See et al., 2013).

#### 2.1.2 Pulmonary regurgitation model

Severe PR was induced in male 250 ± 20 g Sprague–Dawley rats (Charles River, Senneville, Quebec) (*n* = 10–12/group) as previously described (Akazawa et al., 2021; Ebata et al., 2021). Following a midline sternotomy, a partial left‐lobe thymectomy was performed to access the main pulmonary artery. A purse‐string suture was placed on the main pulmonary artery, and a 22G needle was used to repeatedly puncture and lacerate the anterior pulmonary valve leaflet using the bevel of the needle, leading to severe PR without inducing RV outflow obstruction (Akazawa et al., 2021). Chest and skin were closed in layers. Severe PR was confirmed by visualization of a wide regurgitant jet with flow reversal starting in the pulmonary artery branches by echocardiography (Van Berendoncks et al., 2019). One week after surgery, rats were started on 300 mg/kg/day TRN or vehicle (0.5% carboxymethylcellulose), *p.o*., until the terminal experiment at 12 weeks (See et al., 2013). Animals that did not develop PR, despite attempts to do so, were used as surgical sham‐controls (*n* = 6). Of these six shams, four were treated with vehicle and two with TRN. No differences in RV function, fibrosis, and molecular signaling were found between sham groups. Therefore, to increase statistical power while minimizing further sacrifice of animals, data from the two sham groups were combined and analyzed as one group, *n* = 6.

### 2.2 Echocardiography

Rats were anesthetized using 2% isoflurane and supplemented with 100% O_2_. Rats were placed on a temperature‐controlled heated stage (Visualsonics Imaging station, Toronto, ON) to maintain body temperature (37 ± 1°C). The chest was shaven and depilated. Transthoracic echocardiography was performed at the terminal experiment with a 12‐MHz phased‐array probe (Vivid E9, GE Healthcare, Wauwatosa, WI). Two‐dimensional short‐axis views of the RV at the LV papillary‐muscle level and apical 4‐chamber views were acquired. In the apical 4‐chamber view, the end‐diastolic (EDA) and systolic areas (ESA) of the RV were traced and the fractional area of change (FAC) calculated as (EDA‐ESA/EDA x100%) as a measure of global RV systolic function. An M‐mode cursor was placed through the lateral tricuspid annulus to sample tricuspid annular systolic planar excursion (TAPSE) as a parameter of RV longitudinal systolic function. Pulse‐wave tissue Doppler (TDI) was sampled at the lateral tricuspid annulus and peak systolic (S′) and early diastolic (E′) annular velocities were measured as parameters of RV longitudinal systolic and diastolic function, respectively. Pulse‐wave Doppler was used to evaluate tricuspid valve closure‐to‐opening time (TCOT) and pulmonary valve ejection time (ET). The myocardial performance index (MPI) was calculated as (TCOT‐ET/ET) as a combined measure of RV systolic and diastolic function. Data were recorded and analyzed by J.D.D., K.Y., and Y.A. offline (EchoPAC, GE Healthcare, Wauwatosa, WI).

### 2.3 Hemodynamics

Cardiac catheterization was performed after echocardiography. After a median sternotomy, a 2F‐conductance catheter (SPR‐838, Millar Instruments, Inc., Houston, Texas) was advanced through the RV apex. RV peak systolic and end‐diastolic pressures (RVSP and EDP), rate of ventricular pressure rise and decay (+dP/dT and −dP/dT) and time constant of monoexponential decay of RV pressure (𝜏) were obtained during steady‐state measurements. RV contractility (end‐systolic elastance [Ees], preload recruitable stroke work [PRSW]), and chamber stiffness (end‐diastolic pressure‐volume relations [EDPVR]) were determined by analyzing a family of pressure‐volume loops during inferior vena cava occlusion. Injection of hypertonic saline (30% NaCl) was performed to calibrate the parallel conductance of the RV muscle. Blood resistivity was calibrated using Rho cuvettes. Alpha calibration was performed using stroke volume derived from echocardiography. Data were analyzed using LabChart and MVPS Ultra (ADInstruments, Colorado Springs, CO). Rats were euthanized via exsanguination. The heart was harvested and the RV and LV plus septum (LV + S) were separated and individually weighed to calculate the Fulton ratio as a measure of RV hypertrophy. The RV was sectioned and stored by the following: snap‐frozen using liquid nitrogen and stored at −80°C for protein analysis, and another section was fixed in 10% formalin for histology.

### 2.4 RV cardiomyocyte size and fibrosis

A 5 μm microtome section of the RV was stained with hematoxylin–eosin (H&E) (NC1470670, Thermo Fisher Scientific, Massachusetts, USA) or picrosirius red (PSR) (NC9908782, Thermo Fisher Scientific, Massachusetts, USA). Interstitial fibrosis was evaluated by detecting collagen positive areas (red) and calculated by proportional comparison of collagen area to total tissue area (ImageJ). Cardiomyocyte hypertrophy was evaluated from H&E staining by tracing 80–150 cardiomyocytes per animal. The average cardiomyocyte cross‐sectional area (CSA) per animal group was analyzed.

### 2.5 Primary RV fibroblast cultures

RV fibroblasts were derived from juvenile Sprague–Dawley rats (90 ± 10 g). RVs were fragmented and enzymatically digested using a collagenase type II solution (LS0041676, Worthington Biochemical Corp., NJ, USA) to isolate primary fibroblasts (Sun et al., 2018). Isolated cells were cultured in DMEM with 10% FBS and 1% antibiotic‐antimycotic and plated on 100 mm^2^ culture dishes. Cells were incubated and grown in a 37°C and 5% CO_2_ environment. Non‐adherent cells were washed out after overnight incubation. Cells were then passaged and seeded onto 6‐well plates. At 80% confluence, cells were serum‐starved for 2 h and stimulated and/or treated with DMSO (vehicle), AG1295 (658550, Sigma‐Aldrich, St. Louis, USA) (PDGFR inhibitor) at 10 μM, and recombinant TGFβ1 (616461, Sigma‐Aldrich, St. Louis, USA) at 5 ng/mL. After 24 h, wells were washed using 1× PBS and intracellular proteins were collected using lysis buffer (Invitrogen, Grand Island, NY) with 100× PMSF. Sample lysates were sonicated and diluted (1:6) with 6× SDS sample buffer (Invitrogen, Grand Island, NY) for western blot assay.

### 2.6 RV fibroblast proliferation assay

As PDGF can promote mesenchymal proliferation, to investigate the effects of PDGF on RV fibroblast proliferation in response to mechanical stress, isolated cells were seeded on 6‐well laminin‐coated BioFlex® plates (BF‐3001C, Flexcell International, Burlington, USA) and grown to 70% confluence (Sun et al., 2018). Cells were then subjected to 15% cyclical equiaxial stretch at 1.2 Hz for 24 h (FX‐6000T, Flexcell International, Burlington, USA). Non‐stretched (NS) controls were kept in the same incubator. Stretched cells were treated with 10 μM AG1295 or 100 μM TRN. Cells were fixed and permeabilized using cold (−20°C) methanol for 20 min and subsequently blocked in 10% BSA/PBS for 30 min. Lamin‐coated membranes were incubated with primary antibodies for Ki‐67 and vimentin (Sigma‐Aldrich, St. Louis, USA) overnight followed by incubation of appropriate fluorescein‐conjugated secondary antibodies (Sigma‐Aldrich, St. Louis, USA) for 1 h. Nuclei were counterstained using Hoechst stain (Thermofisher Scientific, Waltham, MA) for 5 min. Membranes were washed twice with 1× PBS and mounted onto glass slides with a coverslip using anti‐fade mounting medium (Vectashield, Vecta Laboratoies, Newark, CA). Slides were visualized using a Nikon Eclipse E1000 microscope (Nikon, Melville, NY) and images captured using NIS Elements BRv4.11 software (Nikon, Melville, NY). At 20× magnification, 20–25 random fields were imaged at different regions of the membrane. ImageJ was used to quantify Hoechst stained and Ki‐67 positive nuclei to determine the proliferation index (PI) as (Ki‐67 positive nuclei)/(Hoescht stained nuclei) × 100% (Sun et al., 2018).

### 2.7 Protein analysis (Western blotting)

To extract proteins, frozen RV sections were homogenized using a sterile metal bead and Tissue Extraction Reagent II (1 mL: 50 mg) (Thermofisher Scientific, Waltham, MA) with proteinase‐phosphatase inhibitor. Tissue lysates were diluted 1:1 with 2× SDS sample buffer (Invitrogen, Grand Island, N.Y). In vivo and in vitro protein samples were separated by gel electrophoresis and transferred to a PVDF membrane using a transfer apparatus (Biorad). Membranes were blocked using 5% BSA overnight at 4°C to reduce non‐specific binding. Membranes were incubated with antibodies detailed in Table S1. Densitometry was performed using Image StudioTM Lite (LI‐COR Bioscience, Lincoln, Nebraska). Western blots for PAB experiments are expressed as a fold change relative to sham + vehicle controls to control for variability between blots.

### 2.8 Statistics

Data are expressed as mean ± SD/SEM, as appropriate. Statistical analysis was performed using GraphPad Prism 10. Differences between groups were analyzed by One‐Way ANOVA with Tukey's Multiple Comparisons *post‐hoc* test. Statistical significance was regarded when *p* < 0.05.

## 3 RESULTS

### 3.1 RV and body weight

At protocol onset, rats that underwent sham surgery had lower body weights compared to rats undergoing PAB surgery (Table 1). This difference approximately equates to 5 days in age (~7–10 g/day). Despite this difference, at the terminal experiment, PAB rats had lower body weight versus shams. RV weight and the Fulton index were increased in PAB animals versus shams. The Fulton index was reduced with TRN treatment, indicating reduced RV hypertrophy. Body weights of PR and 12‐week sham groups were similar at protocol onset. At the terminal experiment, TRN‐treated PR rats weighed less than vehicle‐treated controls (Table 1). RV weights and the Fulton index were increased in PR animals versus sham controls. TRN reduced both RV and LV + S weights versus vehicle‐treated PR rats, and the Fulton index did not differ versus 12‐week sham groups.

**TABLE 1:** Animal characteristics.

	Sham + Vehicle (*n* = 12)	Sham + TRN (*n* = 12)	PAB + Vehicle (*n* = 9–12)	PAB + TRN (*n* = 10–12)	12 W Sham (*n* = 6)	PR + Vehicle (*n* = 9)	PR + TRN (*n* = 9)
Surgical BW (g)	201 ± 6	208 ± 10	249 ± 9**	248 ± 17**	237 ± 18	255 ± 11*	246 ± 9
Terminal BW (g)	601 ± 52	564 ± 52	525 ± 41*	518 ± 57**	515 ± 44	577 ± 72	479 ± 32^##^
RV (g)	0.33 ± 0.04	0.3 ± 0.04	0.67 ± 0.14**	0.57 ± 0.11**	0.26 ± 0.054	0.39 ± 0.07**	0.29 ± 0.05^##^
LV + IVS (g)	1.09 ± 0.09	1.0 ± 0.09	1.07 ± 0.15	1.1 ± 0.15	0.92 ± 0.08	1.05 ± 0.13	0.89 ± 0.08^#^
Fulton index (RV/LV + IVS)	0.3 ± 0.03	0.29 ± 0.04	0.63 ± 0.09**	0.5 ± 0.09^††,^ **	0.29 ± 0.04	0.38 ± 0.06*	0.32 ± 0.06

*Note*: Data are expressed as mean ± SD. *p < 0.05, **p < 0.01 versus sham controls; ^†^ *p* < 0.05, ^††^ *p* < 0.01 versus PAB + vehicle; ^#^ *p* < 0.05, ^##^ *p* < 0.01 versus PR + Vehicle.

Abbreviations: 12W, 12‐week; BW, body weight; LV + IVS, left ventricle plus septum; RV, right ventricle.

### 3.2 Hemodynamic loading

**FIGURE 1:**
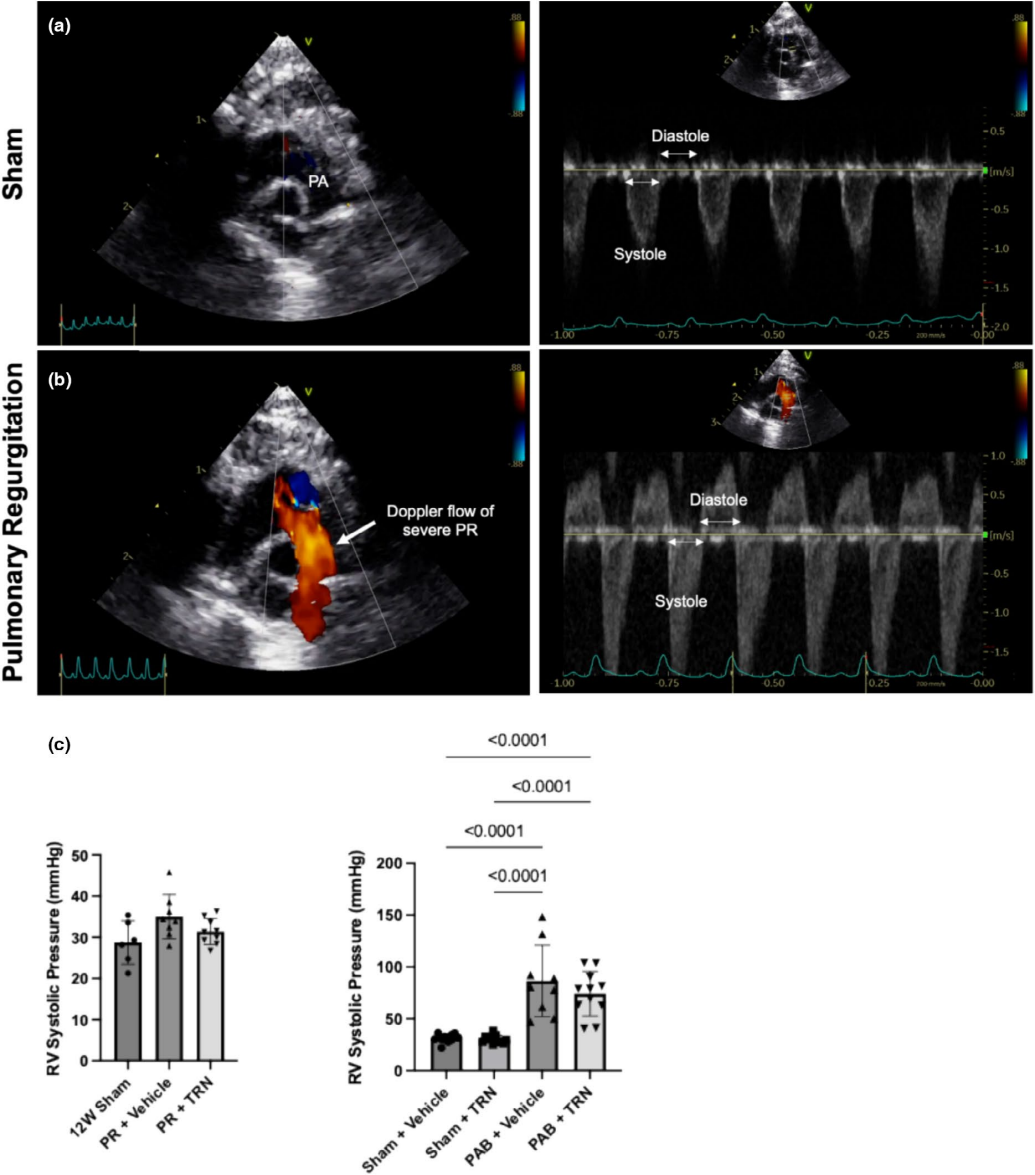
PAB increases RVSPs and PR results in diastolic retrograde blood flow. (a, b) Representative images showing colour flow and pulsed wave (PW) Doppler echocardiography. (a) Depicts a lack of red colour Doppler signal during diastole in sham rats while (b) PR rats show diastolic retrograde blood flow in the main pulmonary artery. Retrograde flow originating from branch pulmonary arteries indicates severe PR. (c) Quantitative analysis of RV systolic pressures measured by conductance catheters in PR, PAB, and sham controls. Graphs depict that PAB induced elevated RV systolic pressures while no differences were found in PR rat groups compared to sham controls. Data are expressed as mean ±SD. PA, pulmonary artery; PAB, pulmonary artery band; PR, pulmonary regurgitation; PW, pulse wave; RV, right ventricle; TRN, Tranilast.

In contrast to sham controls, PR rats displayed retrograde blood flow in the main pulmonary artery, confirmed by color Doppler (Figure 1a,b). No differences in RVSPs were found between PR and 12‐week sham rats (Figure 1c). PAB rats had significantly elevated RVSPs versus controls and were unaltered with TRN treatment (Figure 1c).

### 3.3 RV echocardiography

RV echocardiographic parameters are shown in Table 2. RVEDAi and RVESAi were increased in PAB and PR rat groups compared to age‐matched sham controls. TRN reduced RV size in PAB, but not in PR rats. The reduction in RV chamber sizes with TRN treatment consequently led to a trend in increased RVFAC in PAB rats. RV longitudinal systolic function, as measured by TAPSE and TV S′, was reduced in PAB versus shams and improved with TRN treatment. PR rats exhibited reduced TAPSE relative to 12 W sham controls and were unaltered with TRN. Diastolic relaxation, as reflected by TDI E′, was reduced in PAB and PR rats. TRN treatment led to significantly improved TV E′ in PAB rats, while a trend to improved TV E′ was observed in PR rats as no differences between 12W shams and PR + TRN were observed. PAB rats exhibited worsened global RV function, as reflected by increased RV MPI, and reduced with TRN treatment.

**TABLE 2:** RV function assessed by echocardiography.

	Sham + Vehicle (*n* = 12)	Sham + TRN (*n* = 12)	PAB + Vehicle (*n* = 9–12)	PAB + TRN (*n* = 11–12)	12 W Sham (*n* = 5–6)	PR + Vehicle (*n* = 8–9)	PR + TRN (*n* = 9)
RV EDAi (cm^2^/m^2^)	7.38 ± 0.69	7.27 ± 1.09	12.46 ± 2.86***	10.27 ± 2.21**, †	6.95 ± 0.53	10.41 ± 1.35***	10.46 ± 1.62***
RV ESAi (cm^2^/m^2^)	3.74 ± 0.58	3.59 ± 0.87	8.9 ± 2.51***	6.67 ± 1.91**, †	3.3 ± 0.67	5.98 ± 1.13***	5.78 ± 1.1***
RV FAC (%)	49 ± 11	54 ± 16	29 ± 8***	36 ± 6**	53 ± 7	43 ± 5**	45 ± 4*
TAPSE (cm)	0.31 ± 0.02	0.32 ± 0.02	0.19 ± 0.03***	0.24 ± 0.04***^,†††^	0.29 ± 0.02	0.26 ± 0.02*	0.27 ± 0.03
RV MPI	0.15 ± 0.08	0.2 ± 0.1	0.32 ± 0.19*	0.15 ± 0.12^†^	0.18 ± 0.08	0.23 ± 0.12	0.26 ± 0.11
TV S′	5.47 ± 0.9	5.59 ± 0.65	3.71 ± 0.65***	4.92 ± 1.2^†^	5.59 ± 0.61	4.9 ± 0.9	5.4 ± 0.93
TV E′ (cm/s)	7.1 ± 1.2	8.4 ± 2.4	4.21 ± 0.9***	6.31 ± 2.4*	5.6 ± 1.6	3.4 ± 0.9**	4.6 ± 1.3
TV E (cm/s)	78 ± 20	86 ± 24	80 ± 19	75 ± 19	48 ± 17	51 ± 16	47 ± 15
TV A (cm/s)	68 ± 19	82 ± 35	69 ± 18	76 ± 22	69 ± 12	75 ± 10	68 ± 12
TV E/A	1.15 ± 0.3	1.13 ± 0.4	1.14 ± 0.41	0.94 ± 0.36	0.61 ± 0.03	0.70 ± 0.23	0.65 ± 0.16

*Note*: Values are expressed as mean ± SD, *p < 0.05, **p < 0.01, ***p < 0.001 versus sham controls, ^†^ *p* < 0.05, ^†††^ *p* < 0.001 versus PAB + Vehicle.

Abbreviations: BSA, body surface area; EDA, end‐diastolic area; ESA, end‐systolic area; FAC, fractional area of change; MPI, myocardial performance index; RV, right ventricle; TAPSE, tricuspid annular systolic excursion; TV, tricuspid valve.

### 3.4 RV hemodynamics

Hemodynamic data are shown in Table 3. PAB rats had increased RV contractility (as reflected by +dP/dT, PRSW, and Ees) versus shams, which was unaltered by TRN. PAB rats had increased RVEDP and EDPVR compared to sham controls. TRN treatment did not change EDP but led to reduced RVEDPVR in PAB rats, indicating improved RV compliance. TRN‐treated PR rats had lower RVSP/LVSP ratios compared to vehicle‐treated rats, which may account for the increased +dP/dT in vehicle‐treated PR rats. Tau was increased in PR rats versus sham controls, while no differences between TRN‐treated PR rats and sham controls were found.

**TABLE 3:** RV hemodynamics assessed by conductance catheter.

	Sham + Vehicle (*n* = 12)	Sham + TRN (*n* = 12)	PAB + Vehicle (*n* = 9)	PAB + TRN (*n* = 11)	12W Sham (*n* = 5–6)	PR + Vehicle (*n* = 8)	PR + TRN (*n* = 9)
HR (bpm)	299 ± 29	278 ± 29	277 ± 32	276 ± 30	264 ± 28	250 ± 35	265 ± 23
RVSP (mmHg)	31 ± 4	30 ± 4	83 ± 34***	74 ± 22***	29 ± 5	35 ± 5	31 ± 3
RVSP/LVSP (%)	32 ± 3	34 ± 4	78 ± 23 ***	81 ± 20 ***	38 ± 7	43 ± 6	35 ± 5^#^
EDP (mmHg)	4.9 ± 1.3	5.2 ± 1.4	9.9 ± 4***	8.0 ± 2.4*	6.1 ± 1	7.4 ± 1.3	5.6 ± 1.2^#^
+dP/dT (mmHg/s)	1398 ± 290	1329 ± 320	2943 ± 1486***	2527 ± 807**	1076 ± 332	1375 ± 298*	1310 ± 251
−dP/dT (mmHg/s)	−1039 ± 251	−982 ± 242	−2515 ± 1097***	−2276 ± 841***	−580 ± 177	−717 ± 138	−736 ± 177
Tau (msec)	24 ± 8	25 ± 3	31 ± 17	25 ± 8	0.03 ± 0.01	0.067 ± 0.04*	0.04 ± 0.02
PRSW (mmHg)	16.2 ± 7.2	12.4 ± 6.6	51.9 ± 19***	41.1 ± 16***	16 ± 7	17 ± 10	12 ± 3
Ees (mmHg/μL)	0.045 ± 0.03	0.035 ± 0.014	0.14 ± 0.11***	0.15 ± 0.06***	0.041 ± 0.016	0.054 ± 0.053	0.032 ± 0.01
EDPVR (mmHg/μL)	0.004 ± 0.002	0.006 ± 0.003	0.02 ± 0.017**	0.008 ± 0.004^†^	0.0042 ± 0.0011	0.0046 ± 0.0025	0.0040 ± 0.0017

*Note*: Data are expressed as mean ± SD. *p < 0.05, **p < 0.01, ***p < 0.001 versus age‐matched shams; ^†^ *p* < 0.05, ^#^ *p* < 0.05 versus PR + Vehicle.

Abbreviations: +/−dP/dT, maximum or minimum dP/dT; EDP, end‐diastolic pressure; EDPVR, end‐diastolic pressure volume relation; Ees, end‐systolic elastance; HR, heart rate; LVSP, left ventricular systolic pressure; PAB, pulmonary artery band; PR, pulmonary regurgitation; PRSW, preload recruitable stroke work; RVSP, right ventricular systolic pressure.

### 3.5 RV fibrosis and hypertrophy

**FIGURE 2:**
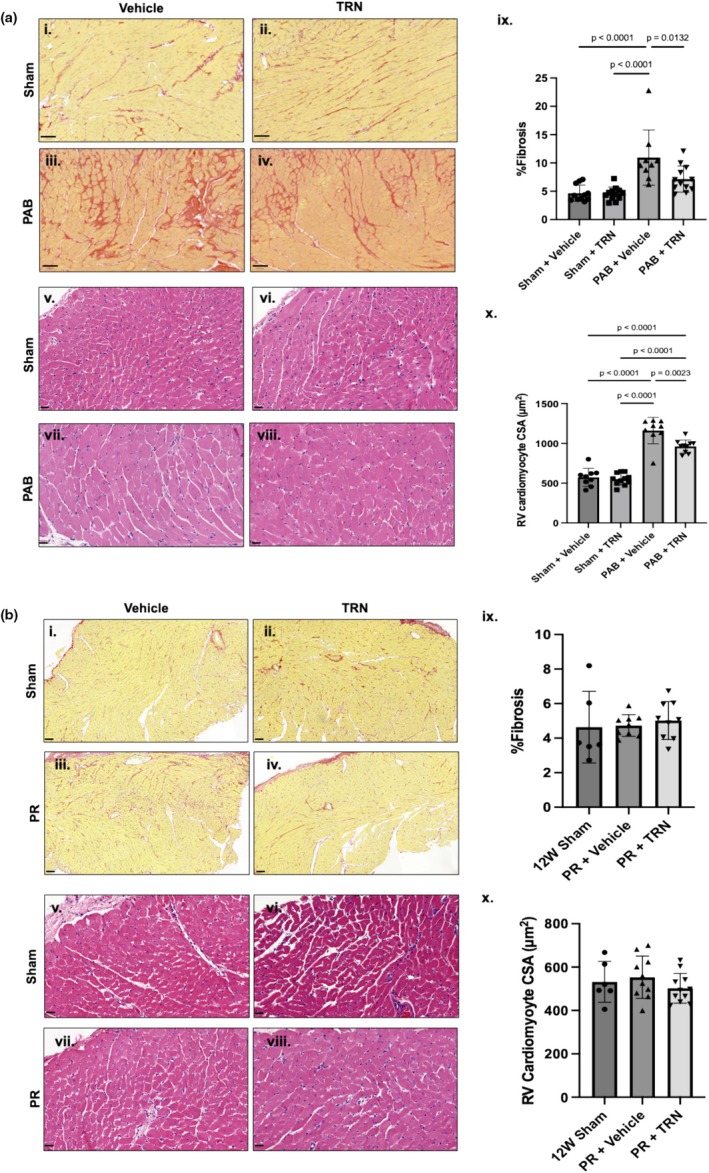
Tranilast reduced RV fibrosis and cardiomyocyte hypertrophy in male PAB rats. (a) PAB rats had increased RV fibrosis (i–iv) and cardiomyocyte hypertrophy (v–viii) compared to sham controls. (b) PR did not induce RV fibrosis (i–iv) or cardiomyocyte hypertrophy (v–viii). TRN treatment reduced RV fibrosis (ix) and cardiomyocyte hypertrophy (x) in PAB. Data are expressed as mean ± SD, CSA, cross‐sectional areas; PAB, pulmonary artery band; PR, pulmonary regurgitation; RV, right ventricle; TRN, Tranilast.

RV collagen deposition and cardiomyocyte hypertrophy, measured by CSAs, were increased in PAB rats versus sham controls and reduced with TRN treatment (Figure 2a). RV fibrosis and cardiomyocyte hypertrophy were not significantly increased in PR rats versus shams (Figure 2b).

### 3.6 Tranilast reduced TGFβ canonical signaling associated with RV fibrosis in PAB and PDGFRβ expression in PR

**FIGURE 3:**
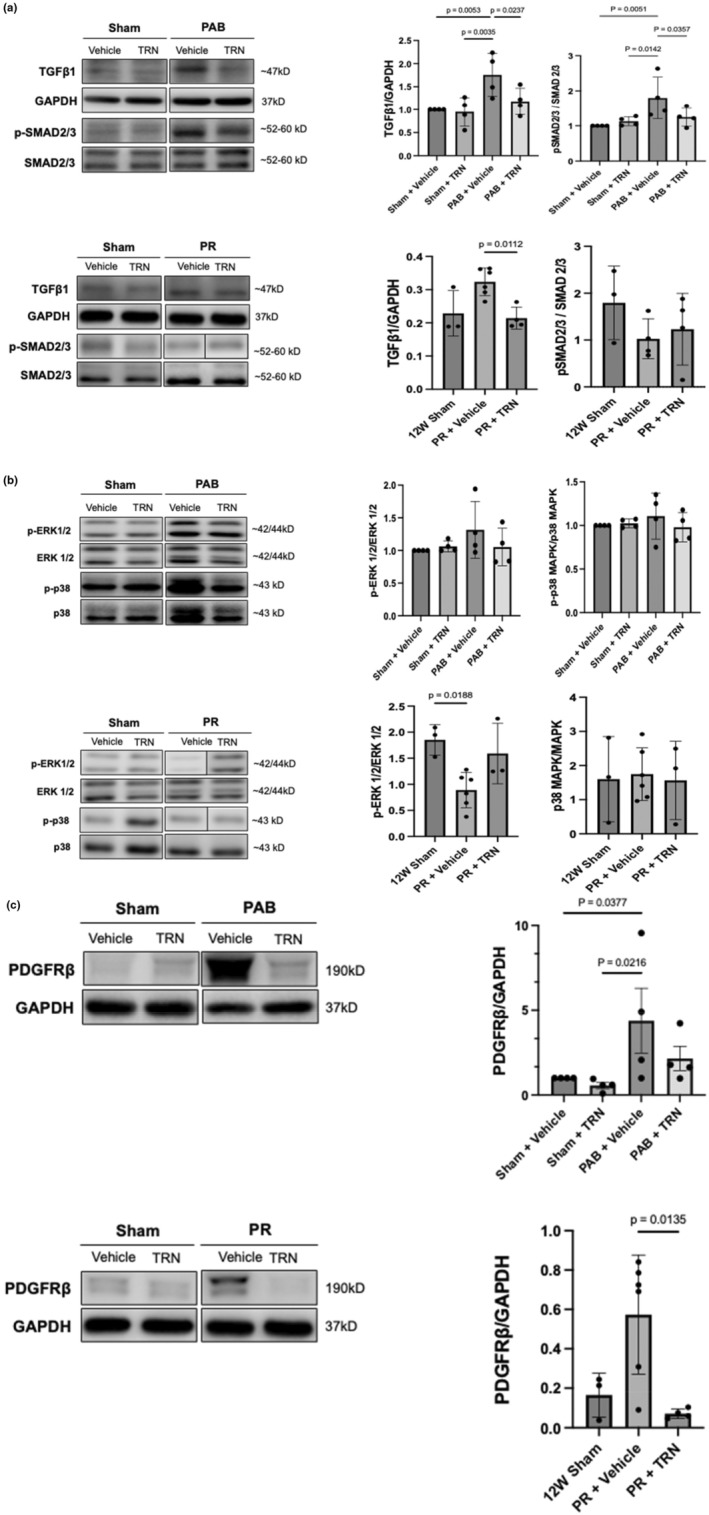
Tranilast reduced TGFβ1 and SMAD signaling associated with RV fibrosis and attenuated PDGFRβ expression in vivo. (a, b) Representative western blots and quantitative analysis of TGFβ1 canonical (a) and non‐canonical signaling (b) and (c) PDGFRβ expression in PAB and PR groups. TRN reduced TGFβ1 expression and SMAD2/3 activity in PAB rats and normalized ERK1/2 activity in PR rats. PDGFRβ expression was increased in vehicle‐treated PAB and PR rats and attenuated with TRN treatment. Data expressed as mean±SD. Sham versus PAB groups are expressed as relative fold‐change relative to sham + vehicle controls, *n* = 9–12/group; *n* = 3–6/group in 12W sham versus PR. 12W, 12 week; ERK, extracellular signal‐regulated kinase; PAB, pulmonary artery band; PDGFRβ, platelet‐derived growth factor receptor β; PR, pulmonary regurgitation; TGFβ1, transforming growth factor receptor β1; TRN, Tranilast.

Representative in vivo western blots and quantitative analyses are shown in Figure 3. TGFβ1 expression and canonical (SMAD2/3) activity (calculated by the ratio of phosphorylated to total SMAD2/3) were elevated in PAB rats versus sham controls and reduced with TRN treatment (Figure 3a). No significant changes were found in TGFβ non‐canonical (ERK and p38 MAPK) signaling in PAB rats (Figure 3b). PAB induced PDGFRβ expression and was attenuated with TRN treatment (Figure 3c). No differences in PDGFRβ expression were found between TRN‐treated PAB rats and sham controls (Figure 3c). PR rats showed trends of increased PDGFRβ expression versus shams, which were significantly reduced with TRN treatment (Figure 3c). TGFβ1 expression was reduced with TRN treatment in PR rats (Figure 3a), while SMAD2/3 and p38 MAPK expression were unchanged in PR rats versus shams. ERK ½ activity was reduced in PR rats versus 12 W sham controls, which reversed to sham levels with TRN treatment, as no differences between PR + TRN and 12 W shams were found (Figure 3b).

### 3.7 PDGF inhibition downregulated SMAD and JNK activity and phosphorylated β‐catenin, which attenuated POSTN expression in RV fibroblasts

**FIGURE 4:**
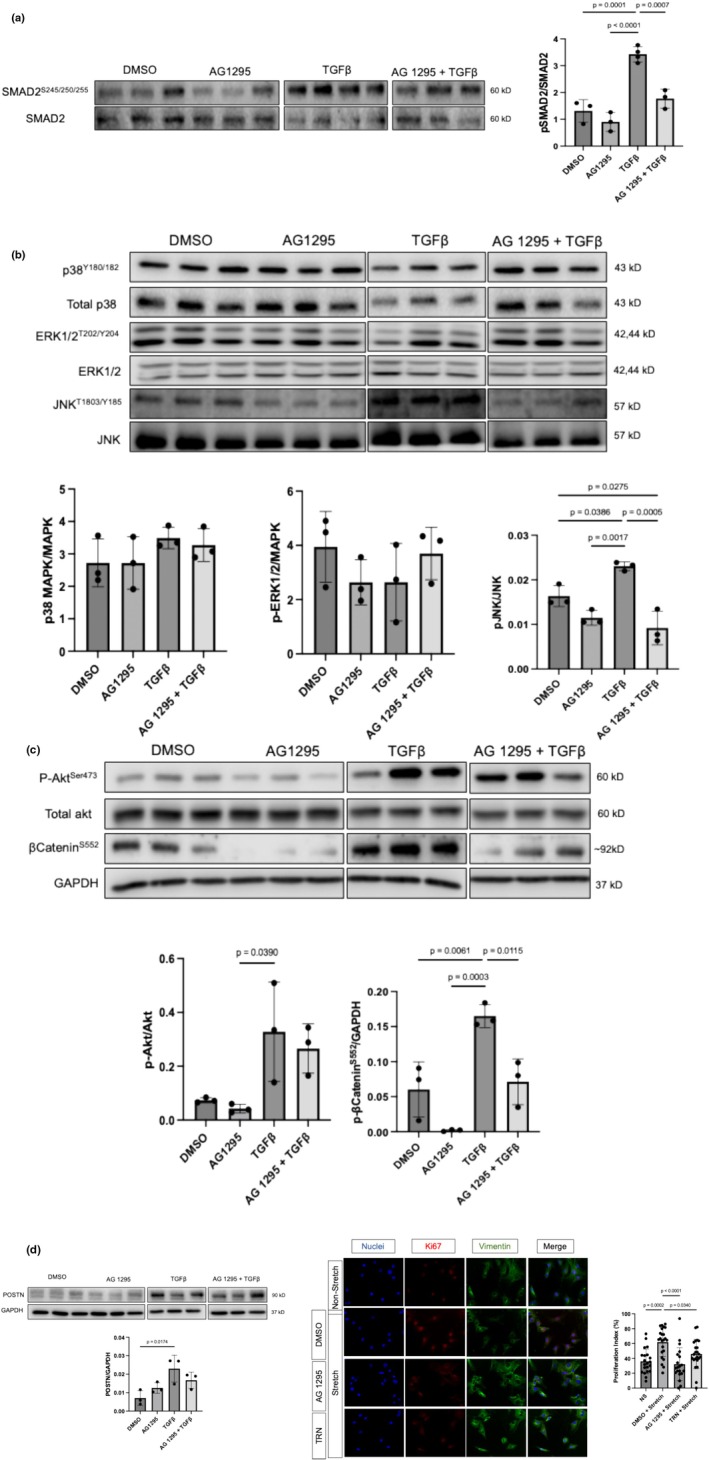
PDGF inhibition reduced SMAD2/3 and JNK activity and β‐catenin phosphorylation to attenuate POSTN expression in TGFβ‐stimulated and reduced RV fibroblast proliferation in mechanically stimulated RV fibroblasts. (a–c) Western blots and quantitative analysis of (a) TGFβ canonical, (b, c) non‐canonical signaling (p38, ERK, JNK MAPKs, and Akt), (c) β‐catenin phosphorylation, and (d) POSTN expression in RV fibroblasts. (d) Representative fields of immunofluorescent stained mechanically stimulated RV fibroblasts to detect proliferating vimentin‐positive cardiac fibroblasts (green), by ki‐67 (red) positive nuclei. Cellular nuclei were stained blue (Hoescht stain). Images are in 20× magnification. Proliferating nuclei are depicted by the overlay of blue and red (purple). Microscopy images are in 20× magnification. Quantitative analysis of the proliferative index (PI), ratio of Ki‐67 positive nuclei to total nuclei. DMSO + stretch (62% ± 19%); AG 1295 + Stretch (32% ± 22%); TRN + Stretch (46% ± 19%). Data are expressed as mean ±SD, NS, non‐stretch; TRN, Tranilast.

To evaluate the crosstalk between TGFβ and PDGF signaling and the effects of PDGF inhibition on RV fibroblast activation, we treated TGFβ1‐stimulated RV fibroblasts with AG1295 and evaluated canonical and non‐canonical components of TGFβ1 signaling. TGFβ1 stimulation induced TGFβ canonical (SMAD2) and non‐canonical (JNK and Akt) activity, reflected by the ratio of phosphorylated to non‐phosphorylated protein, and increased POSTN expression, indicating increased fibroblast activation (Figure 4a–c). Additionally, TGFβ stimulation led to increased phosphorylated β‐catenin (a signaling component of the Wnt canonical pathway) (Figure 4c). AG1295 treatment abrogated SMAD2, JNK, and β‐catenin signaling components and attenuated POSTN expression in TGFβ‐stimulated RV fibroblasts (Figure 4a–d).

### 3.8 PDGF inhibition reduced RV fibroblast proliferation in response to mechanical stress

PDGF signaling mediates mesenchymal cell proliferation in cancer and diseases associated with fibrosis (Bonner, 2004). To investigate the role of PDGF in RV fibroblast proliferation, we tested AG1295 and TRN on mechanically stimulated RV fibroblasts. Twenty‐four hours of a mechanical stimulus led to increased RV fibroblast proliferation compared to non‐stretched controls; a response abrogated by AG1295 and by TRN treatment (Figure 4d).

### 3.9 TGFβ stimulates PDGFRβ expression in RV fibroblasts

**FIGURE 5:**
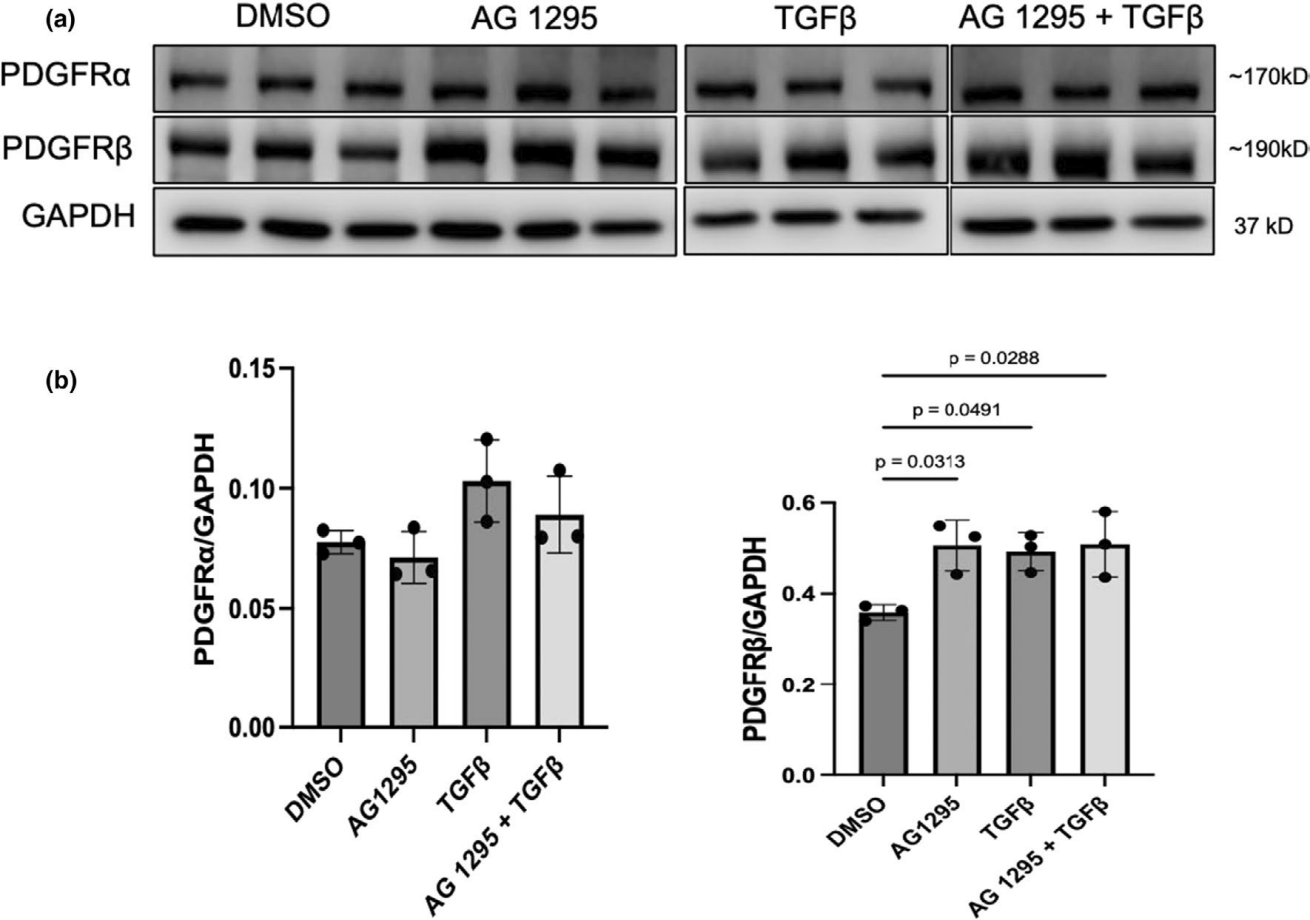
TGFβ1 increases PDGFRβ expression but not PDGFRα. (a) Representative western blots of PDGFRα and PDGFRβ in RV fibroblasts. (b) Quantitative analysis of PDGFRα and PDGFRβ expression. TGFβ1 and AG1295 stimulated PDGFRβ expression, but no significant changes were found in PDGFRα expression. Data expressed as mean ± SD, *n* = 3/group. PDGFR, platelet‐derived growth factor receptor; TGFβ, transforming growth factor β.

To further explain the increased PDGFRβ expression in PAB and PR rats and determine if PDGFRβ expression is present in RV fibroblasts, we evaluated PDGFRβ expression in in vitro stimulated RV fibroblasts. Both TGFβ stimulation and PDGF inhibition induced PDGFRβ, but not PDGFRα, protein expression (Figure 5).

## 4 DISCUSSION

We studied the effects of dual inhibition of TGFβ1 and PDGF on attenuating pathological adverse RV myocardial remodeling in response to RV pressure and volume loading, and the role of PDGF in RV fibroblast proliferation and fibrogenesis. The main results of this study show that:

1. In RV pressure‐loading, TRN reduced TGFβ1 canonical signaling and attenuated PDGFRβ expression, which were associated with reduced RV fibrosis and hypertrophy, and improved RV systolic function and diastolic compliance.
2. In RV volume‐loading, TRN reduced PDGFRβ expression and reversed ERK1/2 to sham levels, which was associated with reduced RV mass and improved RV relaxation, as manifested by improved TDI E′ and tau.
3. PDGF inhibition reduced TGFβ‐stimulated SMAD2/3, JNK, and β‐catenin signaling, resulting in reduced fibroblast activation as reflected by POSTN expression.
4. PDGF inhibition and TRN treatment reduced RV fibroblast proliferation in response to mechanical stress.

Taken together, our results show that PDGF is an important driver of RV fibroblast proliferation and activation in the hemodynamically stressed RV and crosstalks with TGFβ1 signaling to mediate RV fibrosis.

### 4.1 Reduced TGFβ canonical signaling is associated with improved RV fibrosis and function in RV pressure‐loading

The relationship between RV fibrosis and RV adaptation and maladaptation is incompletely defined (Crnkovic et al., 2019). Indeed, previous studies, including our own, have found that alleviating hemodynamic stress can improve RV function without reducing RV fibrosis (Akazawa et al., 2021; Fujioka et al., 2022). RV fibrosis may initially be an adaptive mechanism which, when excessive, may contribute to maladaptive remodeling (Gordon et al., 2022). We have also previously shown that in RV pressure‐loading, RV fibrosis plays a detrimental role on RV function (Gold et al., 2020; Yazaki et al., 2025). RV fibrosis in rTOF and PAH is related to adverse clinical outcomes (Cochet et al., 2019; Yamamura et al., 2019). Our data add to growing evidence that RV pressure‐loading induces RV fibrosis via TGFβ1, PDGF, and Wnt signaling which leads to RV systolic and diastolic dysfunction and ultimately right heart failure (Ebata et al., 2021; Ramos et al., 2018; Sun et al., 2018). Consistent with our previous work, PAB induced significant RV fibrosis, hypertrophy, dilatation, and dysfunction (Nielsen et al., 2016; Ramos et al., 2018; Sun et al., 2018). The current study shows that these can be attenuated by dual inhibition of TGFβ1 and PDGF. Importantly, these effects were associated with improved RV compliance (Rain et al., 2016). This is clinically relevant as diastolic function is an important determinant of outcomes in CHD and PAH (Friedberg et al., 2012; Rain et al., 2013). Our results are consistent with the effects of TRN on improving LV compliance in LV pathology and fibrosis (See et al., 2013; Kelly et al., 2007). In addition to its positive effects on diastolic function, TRN treatment improved parameters of RV systolic longitudinal function, including improved TAPSE and TV S′. This is clinically relevant, as in PAH patients, reduced RV longitudinal function is associated with poor outcomes (Driessen et al., 2018). Along with RV hypertrophy, excessive fibrosis reduces cardiomyocyte tension development and ventricular contraction in part by disrupting gap junctions impeding excitation‐contraction coupling (Kusakari et al., 2017). Thus, treatment with a dual inhibitor of TGFβ1 and PDGF reduced RV fibrosis and improved RV adaptation and remodeling in response to a continuing pressure‐load.

TRN has been shown to inhibit mast cell cytokine release, macrophage infiltration, and NLRP3 activation (Huang et al., 2018; Kelly, 2004; Mamazhakypov et al., 2024). These inflammatory responses are potential mediators of RV fibrosis and pathological remodeling in response to chronic RV pressure overload (Sydykov et al., 2018). However, we found that MCP‐1, a mast cell‐derived chemokine and a key regulator of macrophage infiltration, was not upregulated in our PAB rat tissues (Figure S1). Our previous work showed that MCP‐1 and other inflammatory cytokines commonly derived from mast cells and other leukocytes (e.g., IL1β, IL4, MIP‐1⍺, and TNF‐⍺) are primarily upregulated early, at the 1‐week time point, in PAB rats (Yazaki et al., 2025). Thus, the anti‐inflammatory effects of TRN, which could have contributed to improved RV remodeling, may not have been captured in the current study at the 6‐week time point.

### 4.2 Reduced PDGFRβ and RV hypertrophy are associated with improved RV relaxation in RV volume‐loading

RV pressure‐loading led to markedly more RV hypertrophy, fibrosis, and dysfunction than RV volume‐loading in our study (Ebata et al., 2021). In clinical settings, RV volume‐loading is often tolerated for long periods, sometimes decades, before overt RV systolic dysfunction manifests (Reddy et al., 2013). Nonetheless, chronic volume loading can lead to RV failure and is thought to be an important mechanism underlying RV failure in rTOF (Reddy et al., 2013). Consistent with previous work, we found that PR was associated with impaired RV relaxation (Akazawa et al., 2021; Reddy et al., 2013). However, in contrast to previous studies, RV fibrosis was not increased in our PR rats (Akazawa et al., 2021). RV hypertrophy, in and of itself, is associated with impaired RV relaxation in rodent models of PR, early in the disease course, prior to the development of increased RV fibrosis (Reddy et al., 2013). Likewise, changes in the titin N2B/N2BA isoforms increase sarcomere stiffness, thereby affecting RV relaxation and early diastolic filling in RV volume‐loading (Hagdorn et al., 2021).

Our data further delineate the possible role of PDGFRβ and ERK1/2 activity in RV hypertrophic remodeling, as increased PDGFRβ expression and reduced ERK1/2 activity was associated with increased RV mass in PR rats. To date, the role of receptor tyrosine kinases, including PDGFR, in mediating RV hypertrophy has not been well studied. In endothelial cells, PDGFR can modulate ERK1/2 activity directly or via DUSP6, which is a negative regulator of ERK1/2 (Jurek et al., 2009). Downregulation of ERK1/2 is also associated with eccentric hypertrophy, which is characteristic of ventricular volume‐loading (Kehat et al., 2011). In contrast, increased ERK1/2 activity promotes the concentric hypertrophy characteristic of cardiac pressure‐loading (Kehat et al., 2011). Consistent with those studies, TRN normalized ERK1/2 activity in both rat models of RV pressure‐loading and volume‐loading, in association with reduced RV hypertrophy. The trend to decreased ERK1/2 activity may be a consequence of reduced TGFβ1 signaling in PAB rats treated with TRN (Matsumoto‐Ida et al., 2006). In contrast, TRN normalized ERK1/2 activity in association with reduced PDGFRβ expression in our PR rats. Thus, in addition to fibrosis, ERK/MAPK signaling may regulate cardiomyocyte hypertrophy in the hemodynamically stressed RV, providing a potential therapeutic target via two different mechanisms. From a clinical perspective, impaired RV relaxation is associated with reduced exercise capacity (Reddy et al., 2013). Thus, the reduced RV mass and improved RV relaxation in PR rats following TRN treatment are clinically relevant.

### 4.3 MAPK activity in RV fibrosis

Although MAPKs mediate diverse extra‐ and intra‐cellular responses in mesenchymal cells and in cardiomyocytes, their role in RV fibrosis is poorly characterized (Rose et al., 2010). We evaluated ERK1/2 and p38 MAPK activity in vivo, as PDGF‐induced ERK1/2 activity can regulate mesenchymal proliferation in the liver; and fibroblast‐specific deletion of p38α MAPK reduces fibroblast activation in cell cultures and in the heart (Bonner, 2004; Molkentin et al., 2017). However, in contrast to SMAD signaling, ERK1/2 and p38 MAPK activity were not upregulated in the pressure‐or volume‐loaded RV, or in TGFβ‐stimulated RV fibroblasts.

The timing of evaluation may also have affected results as ERK activity in RV fibroblasts may oscillate between active and inactive states (Mutlak & Kehat, 2015). ERK1/2 activity is upregulated within 10 min of TGFβ stimulation in cardiac fibroblasts, whereas we evaluated ERK activity at 24 h to better mimic protein outcomes in the chronic setting (Albeck et al., 2013). In mouse models of PAB and hypoxia‐induced RV hypertension, p38 MAPK inhibitors reduced RV fibrosis and improved RV systolic function (Kojonazarov et al., 2017). Nonetheless, in those studies, significant upregulation of p38 MAPK occurred only in hypoxia‐exposed mice, but not in PAB mice, which is consistent with our results (Kojonazarov et al., 2017). Therefore, further studies are required to evaluate MAPK activity in response to various types of RV stress and at different time points.

### 4.4 PDGF inhibition reduces RV fibroblast proliferation and POSTN expression associated with reduced JNK activity and β‐catenin expression

Cardiac fibroblast proliferation and activation can be stimulated by a wide array of signaling mechanisms and are key events in fibrogenesis (Frangogiannis, 2021). We previously reported that the pressure‐stressed RV shows increased Ki‐67 expression (a marker of proliferation) in RV fibroblasts and is associated with increased RV fibrosis (Sun et al., 2018). Our current results show that PDGF inhibition and TRN treatment can attenuate RV fibroblast proliferation induced by mechanical stimulation. Reduced fibroblast proliferation may, in part, have contributed to the reduced RV fibrosis found in TRN‐treated PAB rats. Additionally, our results suggest that PDGF plays a role in driving RV fibroblast proliferation. Previous investigations have reported that PDGF mediates fibroblast proliferation via JNK and β‐catenin (Duan et al., 2012; Sabapathy et al., 2004). Although the exact mechanism cannot be determined from our study, we found that PDGF inhibition was associated with downregulated JNK activity and β‐catenin expression in our TGFβ‐stimulated cells. Increased JNK activity is associated with mesenchymal cell proliferation in response to cellular stress by activating AP‐1 transcription factors (c‐jun and c‐fos), driving the expression of the cell‐cycle regulators *cyclin D1* and other TGFβ responsive genes (Sabapathy et al., 2004). Phosphorylated β‐catenin at the Ser552 position induces TCF/LEF transcriptional activity, which can also stimulate cardiac fibroblast proliferation (Duan et al., 2012). Additionally, JNK and β‐catenin can cooperatively enhance the transcription of pro‐proliferative genes in malignancy (Lee et al., 2009; Saadeddin et al., 2009). However, our results align more with the role of JNK and β‐catenin in activating RV fibroblasts. Reduced JNK and β‐catenin signaling because of PDGF inhibition in TGFβ‐stimulated RV fibroblasts attenuated POSTN expression. β‐catenin/TCF/LEF transcriptional complex, a component of Wnt canonical signaling, promotes the expression of *COL1A1, POSTN*, hypertrophy, and metabolic dysregulation (Duan et al., 2012). Our results may be particularly relevant as Wnt signaling is more upregulated in the stressed RV versus the LV (Nagendran et al., 2008). Regulation of Wnt signaling by TGFβ signaling in LV fibrosis has been previously reported (Działo et al., 2018). TGFβ‐induced PI3K/Akt signaling can inhibit GSK3β, an upstream negative regulator of β‐catenin, leading to reduced β‐catenin degradation and increased activity (Fang et al., 2007). PI3K/Akt signaling is also thought to act downstream of PDGFR signaling, as PDGF‐induced Akt promotes fibrosis in the liver (Fang et al., 2007). Thus, we anticipated that PDGF inhibition would lead to reduced Akt signaling and reduced phosphorylated β‐catenin. However, Akt activity, reflected by the ratio of phosphorylated Akt to total Akt, was not reduced with PDGF inhibition in the TGFβ‐stimulated RV fibroblasts. Alternatively, JNK signaling may have modulated the phosphorylation status of β‐catenin (Lee et al., 2009; Wang et al., 2017). JNK signaling can lead to the phosphorylation of β‐catenin, and JNK inhibition has been shown to reduce β‐catenin activity in mesenchymal cells (Lee et al., 2009; Wang et al., 2017). Thus, it is possible that reduced JNK activity, as a result of PDGF inhibition, may have led to reduced phosphorylation of β‐catenin in our experiments.

### 4.5 TGFβ1‐JNK signaling axis may modulate TGFβ1‐SMAD signaling axis in RV fibroblasts

Our current findings align with the known role of activated SMAD2/3 in driving myofibroblast transition, increasing expression of α‐SMA and POSTN, and cardiac fibrosis, including increased collagen I expression (Khalil et al., 2017). PDGFRs upregulate SMAD signaling and mesenchymal cell activation in hepatic fibrosis (Lee et al., 2016). Our in vitro results confirm this occurs in TGFβ1‐stimulated RV fibroblasts as PDGF inhibition downregulated SMAD2 activity and attenuated POSTN expression. This suggests that PDGF inhibition, itself, may be beneficial to reduce fibroblast activation in pressure‐loading induced RV fibrosis. However, it remains unclear how PDGF inhibition directly reduces SMAD2/3 activity. Accumulating evidence suggests that TGFβ non‐canonical signaling can influence TGFβ1 canonical pathway activity by modulating the phosphorylation status of the SMAD2/3 complex linker regions (Hough et al., 2012; Zhang et al., 1998). Reduced JNK activity, upon PDGF inhibition, may have reduced SMAD2 activity in our TGFβ1‐stimulated RV fibroblasts. This crosstalk between the TGFβ1 canonical and non‐canonical pathways is further supported by studies showing that SMAD3 contains AP‐1 binding domains which facilitate SMAD3 nuclear translocation and transcriptional activity (Engel et al., 1999).

### 4.6 PDGFRβ expression may be regulated by TGFβ1 signaling and mechanical stress

Despite different phenotypic manifestations between PR and PAB rats, as detailed above, we observed upregulation of PDGFRβ protein expression in both in vivo models, suggesting that various regulatory systems are involved in inducing PDGFRβ expression and its role in RV remodeling. Upregulation of PDGFRβ may be driven by TGFβ1 signaling and/or mechanical stress (Peng et al., 2017). TGFβ1 dose‐dependently induces expression of PDGFRβ via SMAD2 and Sp1 transcription factors (Peng et al., 2017). Consistent with our results, TGFβ1 stimulation increased SMAD2 and PDGFRβ, but not PDGFRα expression in RV fibroblasts, and PAB rats exhibited increased TGFβ1/SMAD signaling and increased PDGFRβ. Upregulation of PDGFRβ protein expression augments the PDGFRβ response as its corresponding ligands, PDGF‐B and PDGF‐D, are concomitantly increased with increased inflammation and fibrosis in the injured heart (Zhao et al., 2011). In contrast, our PR rats had increased PDGFRβ without increased TGFβ1 and SMAD2/3 activity. Integrinβ1‐induced mechano‐activation of FAK can increase PDGFRβ expression and activity independent of PDGF (Manso et al., 2009; Sundberg & Rubin, 1996). FAK expression was increased in our PR rats, which may explain increased PDGFRβ expression (Figure S2). Thus, the mechanical stress imposed on the RV myocardium by hemodynamic loading may directly stimulate PDGFRβ expression in addition to an increase in TGFβ1.

## 5 LIMITATIONS

Multiple processes contribute to RV remodeling in response to pressure‐ and/or volume‐loading, and the precise mechanisms of TRN are incompletely characterized. As discussed, TRN can exert anti‐inflammatory effects, which could have contributed to the fibrotic response in the RV pressure‐loading rat models, but these effects were not characterized here. Despite several attempts, we were unable to evaluate phosphorylated PDGFRβ, evaluate PDGFRβ activity, and specific collagens in our in vivo and in vitro experiments. PDGFRβ and PDGF‐D are also expressed by pericytes and vascular smooth muscle cells, which may influence capillary rarefaction and perivascular fibrosis, which are components of RV remodeling; however, we did not investigate the role of PDGFRβ these specific cell types (Nielsen et al., 2016; Zhao et al., 2011). All animal models have limitations, and the experimental timeline of 6 weeks and 12 weeks in PAB and PR, respectively, is short relative to the clinical timeline of months, years, and decades in rTOF and other conditions. Additionally, our study does not include sex‐specific analyses due to limitations imposed by the COVID‐19 pandemic, which is when experiments were performed. Future studies should consider how TGFβ and PDGF signaling mechanisms differ in females in the presence of hemodynamic load, as the influence of genetic sex differences or estradiol on TGFβ and PDGF signaling is unclear (Wits et al., 2023).

## 6 CONCLUSION

Several effects of TRN administered preventatively suppressed pathological remodeling in PAB and PR rats, albeit through different effectors. TRN reduced RV fibrosis and hypertrophy, resulting in improved RV systolic function and diastolic compliance and relaxation in PAB rats. TRN downregulated TGFβ1–SMAD signaling and PDGFRβ expression in association with reduced RV fibrosis, which could be mediated by reduced fibroblast proliferation and activity. In contrast, TRN improved hypertrophy and relaxation in association with normalized ERK1/2 activity and attenuated PDGFRβ expression in PR rats. We also show that PDGF plays an important role in driving RV fibroblast proliferation and that PDGF signaling intersects with TGFβ1 and Wnt canonical signaling pathways to induce RV fibroblast activation. PDGF inhibition reduced SMAD2/3, JNK signaling, and phosphorylated β‐catenin, which attenuated POSTN expression. Overall, our findings identify that these pro‐fibrotic pathways are interconnected to induce RV fibroblast proliferation and activation and promote RV fibrosis.

## AUTHOR CONTRIBUTIONS

All authors have contributed to the study and conception, and design. J.D.D., G.M., S.K., K.Y., Y.A., and T.M. performed animal experiments and contributed to analysis. J.D.D. performed in vitro experiments and protein analysis. M.S. contributed to western blot analysis; J.D.D. and M.K.F. analyzed data and interpreted results. J.D.D. drafted the manuscript and figures. J.D.D., M.K.F., K.A.C., O.H., R.M.W., and S.P.H. edited and revised the manuscript. M.K.F. conceived and designed the research.

## FUNDING INFORMATION

This work was supported by the Canadian Institute of Health Research (PJT‐16249).

## ETHICS STATEMENT

This study was conducted in accordance with humane guidelines of the American Physiologic Society and approved by the Animal Care Committee of the Hospital for Sick Children (Protocol: #59576).

## Supporting information


Data S1.

## Data Availability

Data are available on request.
