# Supplementary material for: Dual inhibition of TGFβ and PDGF improves RV remodeling and function in response to RV pressure or volume‐loading
Source: Physiol Rep. 2025 May 5;13(9):e70339. doi: 10.14814/phy2.70339 (PMC12051373; doi:10.14814/phy2.70339)
Supplement: Supplementary file 1 — Data S1. [file PHY2-13-e70339-s001.docx]

**Supplemental Material**

**Figure S1. MCP-1 Expression in PAB and sham rats.** (A) Representative western blot for MCP-1 expression in PAB and 6-week sham animals. (B) Quantitative analysis of MCP-1 expression, normalized to GAPDH. Data expressed as mean ±SEM, n=3-4/group. Abbreviations: PAB, pulmonary artery band; TRN, Tranilast.

**Figure S2. FAK expression in PR and 12W Sham Rats** (A) Representative western blot for FAK expression in PR and 12W sham animals. (B) Quantitative analysis of FAK expression, normalized to GAPDH. Data expressed as mean ±SEM, n=6 for 12W sham, n=11 for PR + Vehicle, n=9 for PR + TRN. *p<0.05. Abbreviations: 12W, 12 Week; PR, pulmonary regurgitation; TRN, Tranilast.

**Table S1. List of antibodies used for western blot assay**

| **Antibody** | **Dilution** | **Predicted Molecular Weight (kDa)** | **Catalogue No.** | **Manufacturer** |
| --- | --- | --- | --- | --- |
| Akt | 1:2000 | ~60 | 9252 | Cell Signaling Technology, Boston, MA |
| Akt^Ser 473^ | 1:2000 | ~60 | 4060 | Cell Signaling Technology, Boston, MA |
| CTGF | 1:1000 | ~38 | PA5-32193 | Thermofisher Scientific, Waltham, MA |
| ERK 1/2^Thr202/Tyr204^ | 1:1000 | 44/42 | 9101 | Cell Signaling Technology, Boston, MA |
| ERK1/2 | 1:1000 | 44/42 | 9102 | Cell Signaling Technology, Boston, MA |
| FAK | 1:1000 | 125 | 3285 | Cell Signaling Technology, Boston, MA |
| GAPDH | 1:30,000 | 37 | G8796 | Sigma-Aldrich, Oakville, ON |
| JNK | 1:1000 | ~46 | 9252 | Cell Signaling Technology, Boston, MA |
| JNK^Thr183/Tyr185^ | 1:1000 | ~46 | 9251 | Cell Signaling Technology, Boston, MA |
| MCP-1 | 1:1000 | ~15 | AB25124 | Abcam, Cambridge, UK |
| P38 MAPK | 1:1000 | 40 | 9212 | Cell Signaling Technology, Boston, MA |
| p38 MAPK^Thr180/Tyr182^ | 1:1000 | 43 | 9216 | Cell Signaling Technology, Boston, MA |
| PDGFRα | 1:1000 | 170 | SC-398206 | Santa Cruz Biotechnology Inc., Santa Cruz, CA |
| PDGFRβ | 1:1000 | 190 | 3169 | Cell Signaling Technology, Boston, MA |
| POSTN | 1:1000 | ~93 | NBP1-30042 | Novus Biologicals, Toronto, ON |
| SMAD 2/3 | 1:1000 | 60,52 | 3102 | Cell Signaling Technology, Boston, MA |
| SMAD2/3^Ser465/467^ | 1:1000 | 60,52 | 8828 | Cell Signaling Technology, Boston, MA |
| SMAD2^Ser245/250/255^ | 1:1000 | 60 | 3104 | Cell Signaling Technology, Boston, MA |
| TGFβ1 | 1:1000 | 42,25,12 | 3711 | Cell Signaling Technology, Boston, MA |
| β-Catenin^Ser 552^ | 1:000 | 92 | 9566 | Cell Signaling Technology, Boston, MA |
| Goat anti-rabbit IgG-HRP | 1:2000 | N/A | sc-2004 | Santa Cruz Biotechnology Inc, Santa Cruz, CA |
| Goat anti-mouse IgG-HRP | 1:2000 | N/A | sc-2005 | Santa Cruz Biotechnology Inc, Santa Cruz, CA |
